# Bladder hernia: a rare clinical image

**DOI:** 10.11604/pamj.2023.46.47.38892

**Published:** 2023-10-02

**Authors:** Abdessamad El Bahri, Ahmed Ameur

**Affiliations:** 1 Urology Department, Military Instruction Hospital Mohamed V, Rabat, Morocco,; 2 Faculty of Medicine and Pharmacy, Sidi Mohammed Ben Abdellah University, Fez, Morocco,; 3 Sidi Mohamed Ben Abdallah University, Fez, Morocco,; 4 Mohamed V University, Rabat, Morocco

**Keywords:** Bladder hernia, rare pathology, hernia

## Image in medicine

Bladder hernia is a relatively uncommon medical condition, accounting for 0.5% to 4% of all hernias, primarily affecting men between the ages of 50 and 70. It can lead to a range of symptoms, from mild irritative syndrome to severe obstructive renal failure. A noteworthy clinical indicator is the two-stage micturition process, which involves pressure on the hernia and its disappearance after urination. However, in many cases, this condition remains asymptomatic and is typically diagnosed during surgery. Common complications associated with bladder hernia include urinary tract infections, urinary calculi, and the rare occurrence of intrahernial bladder tumors. Treatment involves surgical intervention to repair the hernia and reposition the bladder. The protruding portion can be removed in cases of significant hernia size, bladder diverticulum, short neck, or bladder necrosis. We present the case of a 61-year-old patient with a history of ischemic stroke due to arterial hypertension. The patient sought medical attention for lower urinary tract symptoms, including dysuria, frequent urination (pollakiuria), and a burning sensation during urination (micturitional burns), which had been ongoing for three years. An abdominal computed tomography scan revealed the presence of a bilateral inguinal hernia containing the bladder on the right side (sagittal section and transverse section). The patient underwent bilateral surgical repair of the hernia and bladder reintegration. The postoperative recovery was uneventful.

**Figure 1:**
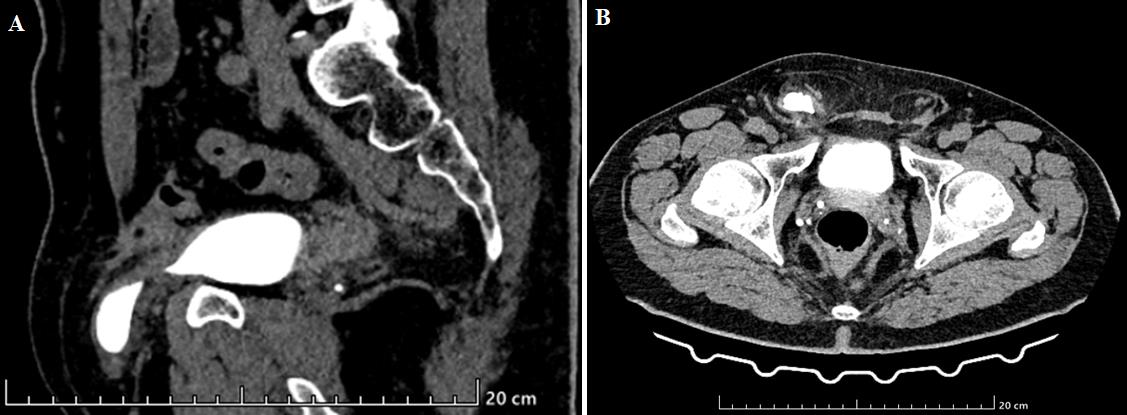
A) sagittal section of the right bladder hernia; B) transverse section of the right bladder hernia

